# Corporate Social Responsibility and Consumer Emotional Marketing in Big Data Era: A Mini Literature Review

**DOI:** 10.3389/fpsyg.2022.919601

**Published:** 2022-05-26

**Authors:** Jing Shao, Tianzi Zhang, Haohui Wang, Yuanhao Tian

**Affiliations:** ^1^Business School, Qingdao University, Qingdao, China; ^2^Financial Department, Qingdao University, Qingdao, China; ^3^Steven J. Green School of International and Public Affair, Florida International University, Miami, FL, United States

**Keywords:** corporate social responsibility, consumer, emotional marketing, big data, performance

## Abstract

In the digital era, big data can strengthen the awareness of corporate social responsibility (CSR) and make CSR more transparent to consumers. While big data continues to deepen the business transformation of enterprises, it is also a process of constantly understanding consumption and public expectations. In this process, the cognitive structure of enterprises is constantly adjusted, no longer simply pursuing performance but constantly realizing the expectations of users and society in order to maintain performance. Through mass media, corporate media, and other platforms, CSR is easier to affect consumers’ emotions. By reviewing the theory of emotional marketing and related research, this paper focuses on the different emotional ties between CSR and consumers and their different effects on consumers. This paper further emphasizes the profound significance of emotional marketing theory for understanding CSR in the era of big data. In addition, this paper also calls for more research based on big data technology, broken down by consumer needs – more specific attention to the different impacts of CSR on different consumers.

## Introduction

In the era of mobile Internet and we media, every move of an enterprise, as long as it violates public social order, will be amplified by netizens and may detonate the network instantly. Therefore, currently, crisis management should be “*ex ante* management,” so that sporadic negative information can be perceived in time: let the potential crisis in the early stage get an early warning; let the trend of public opinion get insight; and make the response of enterprises more scientific and accurate. In addition to fully mastering public opinion big data and establishing analysis models, enterprises must also have a sound sense of social responsibility. While pursuing economic interests and innovation, enterprises should not only gain insight into users through big data but also pay attention to the emotional needs of consumers and the mainstream value of the public. For general enterprises, big data can strengthen the awareness of CSR. The continuous deepening of business transformation through big data is also the process of constantly understanding consumption and public expectations. In this process, the cognitive structure of enterprises is constantly adjusted, not only to pursue performance but also to constantly realize the expectations of users and society in order to maintain performance. Enterprises with a high sense of social responsibility will be able to attract consumers and win market share for a long time. CSR is no longer a halo attached to commercial interests or a passive response to external expectations but the core component of corporate competitiveness and the long-term goal of active pursuit.

There are challenges in operating a strategic planning process that is situated in an uncertain, volatile, and dynamic business environment. It requires an integrated partnership between the inter-organizational actors and other stakeholders. Social media is the integrator of resources and CSR helps to build relationships, acting as a reinforcer of trust (Bowen et al., 2020). López-Fernández and Silva (2021) analyzed the big data retrieved from Twitter related to five firms that have started to be socially responsible but have yet to obtain stakeholders’ legitimacy granted by the engagement in corporate social responsibility. The article contributes to the understanding and effects of firm dynamics in corporate social responsibility or lack thereof, on social networking sites by means of big data analysis. Barbeito-Caamaño and Chalmeta (2020) show an alternative method to evaluate sustainable development and CSR practices based on the opinions of corporate stakeholders expressed on Twitter. Knowledge about CSR practices and stakeholders’ opinions can be obtained using big data to analyze CSR information about the firms on online social networks. These findings have benefits for the stakeholders, who will be able to know the CSR practices of a firm in a more objective way, and for the firm, which will be able to improve its CSR performance and communication strategy, as well as the stakeholder engagement. She and Michelon (2019) examine stakeholders’ perceptions of CSR disclosures by exploiting big data about the interactions between firms and stakeholders in social media. They draw on organized hypocrisy theory to explore how stakeholders react to hypocrisy talk, decisions, and actions strategies employed in CSR disclosures on Facebook. The findings show that the use of organized hypocrisy disclosure strategies in social media allows firms to manage stakeholder perceptions and maintain legitimacy. Companies increasingly recognize the importance of communicating CSR including their engagement with employees, the community, the environment, and other stakeholder groups to attract applicants. Jakob et al. (2021) scraped 67,189 posts published on corporate Facebook career pages by 58 Fortune 500 companies from the time they began their respective career page until June 2018. The results show that a low CSR performance strengthens the effect of CSR communication on organizational attractiveness. Thus, inconsistencies between CSR communication and CSR performance seem to lead to positive evaluations among applicants.

Emotional marketing is a marketing method that appropriately mobilizes the universal human emotions in the process of marketing activities so as to enhance the brand and products to have more resonance with consumers. All marketing activities that can attract, utilize and stimulate consumers’ positive emotions belong to emotional marketing. The concept of socially responsible marketing is sometimes seen as an extension of CSR. Recognizing that CSR activities will affect various stakeholders, including stakeholders, CSR is promoted as a business model to help the company conduct self-regulation.

At present, big data technology not only promotes the scale and popularity of enterprises but also makes the image of CSR more transparent. Therefore, CSR has attracted more and more attention from consumers. It seems that CSR does not directly affect the “price” and “product” concerned by consumers, but psychology and behavior explain this phenomenon through emotional marketing theory. Emotional marketing points out that feelings build a bridge between CSR and consumers. This paper will first trace back to the interpretation of emotional marketing theory on CSR and consumer relationships in the following part. According to the theory of emotional marketing, CSR is a part of emotional marketing. CSR connects with consumers through their specific emotions. Secondly, this paper arranges and discusses the existing literature. Based on “emotion,” this paper subdivides the current decentralized CSR – emotional marketing theory into gratitude and identity and further discusses the specific relationship between social responsibility and emotional marketing.

This paper has three main research contributions. First, it emphasizes the importance of CSR to emotional marketing theory. With the help of emotional marketing, CSR can better help enterprise marketing. Second, this paper further subdivides and classifies the current scattered literature. Based on most of the current research on CSR emotional marketing, this paper points out that the specific emotional bond between consumers and CSR is gratitude and identity. Thirdly, this paper finally discusses the future research trend in this field. In the conclusion part, this paper pays attention to the shortcomings of the current literature, further calls on scholars to pay attention to the trend of big data, and points out the future research direction.

## Method

This paper combs the relationship between emotional marketing, CSR, and consumers from the following aspects. First, it focuses on the impact of CSR as an essential means of emotional marketing on consumers’ psychology, emotion, identity, value judgment, and decision-making, that is, combing the impact relationship between CSR and emotional marketing from the perspective of perception and response. Second, according to “emotion,” the current decentralized CSR emotional marketing theory is divided into two dimensions: gratitude and identity, and further discuss the perceived relationship between consumers’ CSR based on gratitude and identity.

## Results

### Corporate Social Responsibility and Emotional Marketing – Perception and Response

The significance of emotional marketing for CSR is to connect “CSR” and “consumers,” and make “CSR” a factor of marketing. Consoli and Patrut (2010) pointed out that emotional marketing studies how to stimulate people’s emotions and urge them to buy specific products/services. Further, Consoli and Patrut (2010) explained that emotion is a psychological and physiological state related to a variety of feelings, thoughts, and internal (physical) or external (social) behaviors. Human emotion and cognition are closely linked. In this way, emotion is significant in social behavior. It can stimulate cognitive processes and formulate strategies. Recent research shows that purchase choices and decisions as social actions result from careful analysis of rationality and emotion. Besides, the psychological literature recognizes that emotional status affects every decision-making stage in the purchase process, so emotion plays a crucial role in any social or business decision-making. Consoli and Patrut (2010) believe that customers’ purchase decisions are driven by two needs: the functional needs satisfied by-product functions and the emotional needs related to the psychological aspects of product ownership. Especially in a saturated market, consumer desire is more important than the traditional factors that determine the marketing results, such as demand and supply. In this case, mentality, emotion, and compassion dominate. In addition to quality and price, consumers also need trust, love, and dreams (intangible factors). Therefore, with the emergence of the principle of consumer pleasure, emotion becomes more and more critical. Therefore, these products should have the functions that consumers need and release the feelings that consumers need. Roberts (2004) and Thompson et al. (2006) confirmed that some brands’ emotional marketing strategies apply this strategy: consumer-centered, relational, and storytelling, to establish a deep and lasting emotional bond between consumers and brands. Therefore, when consumers are emotional about CSR behavior, emotional marketing connects “CSR” and “consumers.”

Corporate social responsibility is indispensable for emotional marketing. CSR can provide consumers with three forms of value: emotional value, social value, and functional value, and each of them will enhance or reduce the overall value proposition of consumers. Wisker et al. (2019) analyzed Airbnb, a big data e-commerce platform, and showed that the owner’s CSR information affects the purchase intention. Consumers prefer socially responsible owners. The reason is that companies practicing CSR can improve their reputation so as to attract and retain customers in the long term to improve the company’s performance (Romani et al., 2013).

Furthermore, Aaker (2004), Marin et al. (2009), Skinner et al. (2010), Dennis et al. (2016), and Xie et al. (2019) discussed the different roles of different CSR in emotional marketing. On the one hand, CSR can be used as an effective emotional marketing tool to compete and maintain a competitive advantage in the current rapidly changing and highly competitive environment. Aaker (2004), Chahal and Sharma (2006), Skinner et al. (2010), Xie et al. (2019), and Dennis et al. (2016) pointed out that positive moral emotions and attitudes play an intermediary role in influencing CSR behavior on brand advocacy behavior. In this case, a solid corporate image brings emotional and functional benefits because it helps customers become more attached to the company, thus creating a sense of attachment and emotional bonds. Bhattacharya and Sen (2003) takes the higher education industry as an example. If a university ranks high in the university ranking, its stakeholders will identify it as a leading institution in the field. Students or parents who think the university is good through the university ranking will have a more positive feeling about the institution, such as learning one of the projects more confidently and actively. Similarly, 2019 coronavirus disease research to solve the problems of their local communities, provide financial assistance to students during the COVID-19 epidemic, or work with volunteer programs, which can create emotional connections with their stakeholders. Therefore, in the context of higher education, when stakeholders believe that higher education has a strong corporate image, they will have an emotional attachment to the institution.

On the other hand, CSR is a new and growing financial risk factor. Ogrizek (2002) said that if the company is mismanaged, its reputation may be seriously damaged and may directly negatively impact its business and bottom line. Brown and Dacin (1997), Sen and Bhattacharya (2001), and Lichtenstein et al. (2004), and Marin pointed out that a large number of studies have empirically proved that CSR initiatives enhance consumers’ evaluation and recognition of the company. In turn, these mediating factors will affect consumers’ behavior, resulting in positive relationship results, such as loyalty, positive word-of-mouth, and attachment.

Generally, the above discussions highly recognize the importance of CSR: CSR plays an increasingly important role in marketing, and CSR is an indispensable part of emotional marketing theory. However, in marketing practice, what kind of emotion does CSR stimulate consumers? At present, there are different opinions in the literature. Based on the view that emotional marketing needs to find more accurate emotional ties according to different customer groups (Grappi et al., 2013), this paper classifies the arguments in the existing literature into gratitude, and identity and discusses these emotional ties connecting consumers and CSR in the next part.

### Corporate Social Responsibility and Emotional Marketing – Gratitude

The sustainable survival of the enterprise needs the support of all stakeholders. The enterprise should seek their support and obtain recognition by adjusting its own behavior. In this process, enterprises will respond to the requirements put forward by various pressure groups and stakeholders and take actions to solve some social problems, that is, social responsibility. It is noteworthy that the corresponding relationship between the contribution of enterprises to social welfare and personal moral values will arouse people’s gratitude to these enterprises. Ancient Greek philosophy believed that the impulse to repay good with good might constitute an action tendency to confirm a person’s altruistic values and support a person’s source of gratitude. McCullough et al. (2001) defined gratitude as a positive emotion, which usually comes from the realization that one person benefits from the intentional and thoughtful actions paid by another person. Bartlett and DeSteno (2006) believed that gratitude promotes social relations by supporting reciprocal and prosocial behaviors between benefactors and beneficiaries. Gratitude is often generated when people are beneficiaries of donor prosocial behavior. People are most grateful when they see that the actions of a philanthropist have improved their lives. Amsami et al. (2020) argued gratitude regulates people’s response to the benefits received by encouraging them to give verbal and non-verbal recognition and to provide benefits to their benefactors. In this way, it is not difficult to find that CSR helps to promote the gratitude reciprocity relationship between consumers and enterprises. Generally, CSR will gain a positive reputation from consumers and enhance consumers’ expectations of participating in some advocacy behaviors. In the relationship between consumers and enterprises, strengthening enterprises’ efforts to improve consumer welfare, such as charity activities to produce gratitude, leads to the enhancement of consumers’ commitment to enterprises in the form of reciprocal behavior based on gratitude (Morales, 2005; Palmatier et al., 2009). That is, if a business invests resources for the benefit of consumers, consumers may be grateful and may then repay by increasing trust and commitment to the organization (Park et al., 2016). Therefore, consumers’ gratitude and subsequent desire for return will lead to a commitment to enterprises. Especially for those with strong empathy, Xie et al. (2019) said that those with strong empathy are more likely to give feedback under the influence of gratitude than those with weak empathy. On the one hand, gratitude for CSR will encourage consumers to feedback more valuable information to enterprises. The survey of Grappi et al. (2013) found that consumers are more willing to give positive feedback on CSR activities of active enterprises: for example, try more different products of the company and forward them on new media platforms (such as Twitter and Facebook). On the other hand, gratitude also regulates consumers’ responses based on CSR feelings, such as positive word-of-mouth, resistance to negative information, recognition of the company, and investment in the company (Xie et al., 2015). It is worth noting that not all CSR can arouse the gratitude of stakeholders. Take donation as an example. As a CSR, when consumers perceive the interest motivation of corporate donation, the donation cannot improve the corporate image. Although for irresponsible enterprises, the unconditional donation can help them gain reputation because donation helps such enterprises recover some reputation (Dean, 2003). However, for ordinary enterprises, a utilitarian donation has little effect on maintaining customer relations because people know that this behavior of enterprises is to increase their profitability. Fortunately, CSR alleviates the retaliatory behavior of consumers for the failure of the company’s products because the gratitude of CSR drives consumers to reduce retaliation for the failure of the company’s products. Kim and Park (2020) also noted that the higher the CSR performance (e.g., more public recognition of CSR or CSR frequency), the more significant the buffer effect. Indeed, the attitude of enterprises also acts as a mediator. However, the further model analysis points out that the attitude of enterprises still inspires consumers’ gratitude (Xie et al., 2015). The feedback of enterprises to society and the gratitude of consumers are the dual social response of enterprises and consumers.

### Corporate Social Responsibility and Emotional Marketing – Identity

Identity was initially developed in the field of social psychology and organizational behavior. It meets the needs of social identity and self-definition. In turn, it has been proved to fully regulate the impact of perceived corporate identity on product utilization (Ahearne et al., 2005) and loyalty (Mael and Ashforth, 1992). In fact, for most corporate marketing work, CSR seems to be an almost perfect tool; If done well, it can form a strong and lasting identity-based bond between important stakeholders in all walks of life and the company. More importantly, through internal communication and involving employees in CSR activities, managers will successfully promote the consistency between the corporate identity perceived by external stakeholders (i.e., corporate reputation) and internal stakeholders (i.e., perceived identity).

First, enterprises can increase their moral identity and utilization of CSR products. Moral identity refers to the self-schema organized around a series of moral traits, including caring for others, compassionate, fair, friendly, generous, helpful, diligent, honest, and kind (Aquino and Reed, 2002). He et al. (2016) and Reed et al. (2007) believe that enterprises can stimulate consumers’ moral identity in their publicity activities (for example, by emphasizing the moral relevance of their career-related activities) because previous studies have shown that consumers’ moral identity can be temporarily stimulated/enhanced through moral related exercise and stimulation. Therefore, an important meaning of purchasing a marketing sponsor for brand-related reasons is its impact on whether consumers regard themselves as moral individuals. Moreover, it is worth noting that Sheikh and Beise-Zee (2011) and Vitell (2015) more specifically pointed out that since almost every consumer’s understanding of identity is unlikely to be consistent, different CSR activities have different emotional marketing effects on different consumers. Although good CSR has a similar positive impact on customer attitude, its positive impact is more significant for “customers with high causality.” Sheikh and Beise-Zee (2011) for some products, CSR is mainly to arouse the value recognition of interested consumers. Otherwise, if consumers are not stakeholders, CSR may not help marketing. For example, consumers usually like green products, but when the cost of green products is too high, the same consumer may think that saving money is more ethical than “buying green products” from a personal point of view. Take tourism as an example, Tran et al. (2018) pointed out that all dimensions of destination social responsibility, including economic, environmental, legal, moral, and charitable responsibilities, have significantly improved tourists’ moods, but only legal, moral and charitable responsibilities directly affect tourists’ satisfaction.

In addition, Vitell (2015) pointed out consumers’ reactions to “unethical behavior” - that is, immoral or negative CSR. It is found that the company is not responsible for the mediation of consumers’ negative emotions (i.e., the company is not responsible for the mediation of consumers’ negative emotions), which is a strong mediator of consumers’ negative emotions. Specifically, those who (a) have stronger values of social justice, (b) are more empathetic, (c) have a high core of moral identity, and (d) have a strong relationship or collective self are more likely to experience negative moral emotions such as contempt, anger, and disgust when exposed to the irresponsibility of the corporate environment. After being exposed to negative CSR information, the personal response is not only to spread negative word-of-mouth and complain to the company but also to protest and boycott different authorities. Concerning the positive social responsibility activities of enterprises, personal responses are not only positive word-of-mouth, resisting negative information, and identifying with the company, but also considering investing in the company’s shares. This shows that consumers’ moral feelings can lead to strong participation in actions against or supporting key companies and span multiple fields. In the long run, Vitell (2015) concludes that “good ethics” is “good business.” Although, in the short term, people believe that it is possible to achieve success by cheating consumers, in the long run, this strategy tends to be self-defeating. If an organization is ethical (or “good”), it treats its consumers and other stakeholders fairly. This often benefits companies because consumers will continue to buy products from companies that treat them fairly. No one wants to be deceived. On the contrary, “bad morality” is often “bad business,” especially in the long run, because consumers do not want to “deal with” organizations they do not trust.

Second, based on the above literature, Pirsch et al. (2007), Marin et al. (2009), and Pérez and Bosque (2015) believe that good CSR improves customers’ moral identity, thus increasing customers’ loyalty to corporate brands. Marin Ruiz and Rubio found that when CSR impacts consumer loyalty, identity significance plays a crucial role in this impact. Although a corporate identity may not be consistently significant, companies that activate specific consumer social identities will influence consumers’ responses to product stimuli and increase consumer loyalty. Marin et al. (2009), Pérez and Bosque (2015) collected the information of 1,124 banks serving customers through the test and further confirmed that the CSR image positively impacts customers’ recognition of the company, emotions, and satisfaction caused by the company. This sense of identity will also make customers’ emotions and satisfaction at the institutional level so as to improve customers’ loyalty behavior (Pérez and Bosque, 2015). Although customers sometimes doubt the motivation of CSR projects, “good CSR promotion” could dilute customers’ doubts (Pirsch et al., 2007).

## Conclusion

Taken together, a mini literature review allows us to understand the influential relationship between CSR, emotional marketing, and consumer psychology from a new intersection. In the past, CSR and marketing were two separate components, and most CSR was rarely perceived directly by consumers. But now, more and more companies are taking the initiative to fulfill CSR while also using it as an effective means of emotional marketing. CSR is an important part of emotional marketing theory and consumer psychology can better connect the two. With the development of big data platforms, CSR will be more important. On the one hand, big data has increased CSR exposure among consumers. Massive data characterize big data. Nowadays, many media collect data through big data. CSR is no exception - once the media displays various CSR of enterprises in front of consumers through big data, enterprises have to consider their social image. On the other hand, it also helps enterprises collect consumers’ feedback on CSR. With big data, enterprises can collect more consumers’ feedback on enterprise CSR and more accurately match different consumers’ feedback on CSR more conveniently. The research of Sheikh and Beise-Zee (2011) and Vitell (2015) provides a direction for the subsequent development of emotional marketing. Sheikh and Beise-Zee (2011), and Vitell (2015) focused on the diversity of consumer identity. In fact, not only identity but diversity is also essential for other emotional ties of emotional marketing: people may appreciate CSR, but the specific “which consumer” will appreciate “which CSR” needs to be further studied. Therefore, this paper calls on more scholars to pay attention to CSR emotional marketing and meta-analysis of the response of different consumers’ different emotions to different CSR.

## Author Contributions

JS, TZ, HW, and YT wrote the initial drafts, reviewed the manuscript, and provided comments and feedback. All authors contributed to the article and approved the submitted version.

## Conflict of Interest

The authors declare that the research was conducted in the absence of any commercial or financial relationships that could be construed as a potential conflict of interest.

## Publisher’s Note

All claims expressed in this article are solely those of the authors and do not necessarily represent those of their affiliated organizations, or those of the publisher, the editors and the reviewers. Any product that may be evaluated in this article, or claim that may be made by its manufacturer, is not guaranteed or endorsed by the publisher.
